# The effect of surgical approach in total hip replacement on outcomes: an analysis of 723,904 elective operations from the National Joint Registry for England, Wales, Northern Ireland and the Isle of Man

**DOI:** 10.1186/s12916-020-01672-0

**Published:** 2020-08-06

**Authors:** Ashley W. Blom, Linda P. Hunt, Gulraj S. Matharu, Michael R. Reed, Michael R. Whitehouse

**Affiliations:** 1 Musculoskeletal Research Unit, Translational Health Sciences, Bristol Medical School, 1st Floor Learning & Research Building, Southmead Hospital, Bristol, BS10 5NB UK; 2 National Institute for Health Research Bristol Biomedical Research Centre, University Hospitals Bristol NHS Foundation Trust and University of Bristol, Bristol, UK; 3 Northumbria Healthcare NHS Foundation Trust, Department of Trauma and Orthopaedics, Wansbeck General Hospital, Woodhorn Lane, Ashington, NE63 9JJ UK

**Keywords:** Hip replacement, Surgical approach, Outcomes, Revisions surgery, Mortality

## Abstract

**Background:**

Total hip replacement (THR) is clinically and cost-effective. The surgical approach employed influences the outcome; however, there is little generalisable and robust evidence to guide practice.

**Methods:**

A total of 723,904 primary THRs captured in the National Joint Registry, linked to hospital inpatient, mortality and patient-reported outcome measures (PROMs) data with up to 13.75 years follow-up, were analysed. There were seven surgical approach groups: conventional posterior, lateral, anterior and trans-trochanteric groups and minimally invasive posterior, lateral and anterior. Survival methods were used to compare revision rates and 90-day mortality. Groups were compared using Cox proportional hazards and Flexible Parametric Survival Modelling (FPM). Confounders included age at surgery, sex, risk group (indications additional to osteoarthritis), American Society of Anesthesiologists grade, THR fixation, thromboprophylaxis, anaesthetic, body mass index (BMI) and deprivation. PROMs were analysed with regression modelling or non-parametric methods.

**Results:**

Unadjusted analysis showed a higher revision risk than the referent conventional posterior for the conventional lateral, minimally invasive lateral, minimally invasive anterior and trans-trochanteric groups. This persisted with all adjusted FPM and adjusted Cox models, except in the Cox model including BMI where the higher revision rate only persisted for the conventional lateral approach (hazard rate ratio (HRR) 1.12 [95% CI 1.06,1.17] *P* < 0·001) and trans-trochanteric approaches (HRR 1.48 [95% CI 1.14,1.91] *P* = 0.003). PROMs demonstrated statistically, but not clinically, significant differences. Self-reported complications were more frequent with the conventional lateral approach, and the risk of 90-day mortality was higher (HRR 1.15 [95%CI 1.01–1.30] *P* = 0.029).

**Conclusions:**

Lateral approaches for THR are associated with worse outcomes, including more deaths and revisions, than the posterior approach. We recommend the posterior approach should be considered the current standard approach for THR. Large well-designed studies are needed to assess any potential benefits from using minimally invasive posterior approaches and the conventional anterior approach.

## Introduction

Total hip replacement (THR) is a common operation with low revision rates [1], excellent patient-reported outcome measures (PROMs) [2] and low mortality [3]. Efforts to improve outcomes focus on specific factors, such as implants [4] and thromboprophylaxis [5]. Surgical approach is a relatively simple way to effect outcomes. Although attention has been given to surgical approach over the last decade (e.g. use of mini-incisions, and more recently the anterior approach) [6, 7], there is a lack of well-designed studies comparing outcomes when using different hip approaches.

For THR, the hip can be approached anteriorly, laterally, posteriorly or by detaching the greater trochanter (trans-trochanteric). Each approach can be performed through a limited (minimally invasive) incision/s, although this is extremely rare for the trans-trochanteric, leaving seven common approaches. The anterior approach is intermuscular, the lateral passes through the major hip abductors and the posterior through the short external rotators. The type and extent of soft tissue damage and bleeding caused by each approach differs and thus influences outcome [8–11].

Using data from the National Joint Registry (NJR) for England, Wales, Northern Ireland and the Isle of Man, we compared implant survivorship, PROMs and post-operative mortality between the seven common surgical approaches used for primary THR.

## Methods

**Fig. 1:**
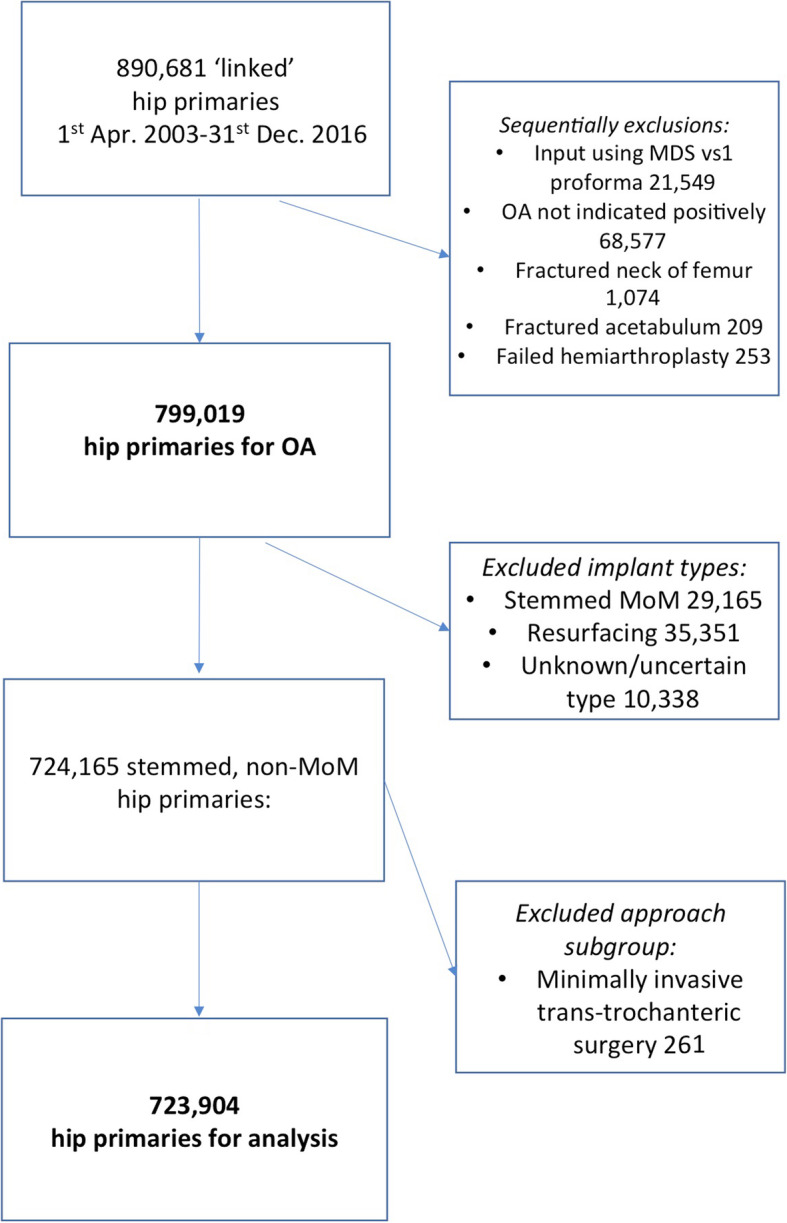
Study selection criteria. Abbreviations: OA = osteoarthritis; MoM = metal-on-metal

Our initial cohort comprised 890,681 linked THRs, performed between 1 April 2003 and 31 December 2016 [12]. We excluded 21,549 (2·4%) where data was collected on version 1 of the minimum data set collection form (MDS v1) that combined the anterior and anterolateral approach. Of the remaining 869,132, we selected 800,555 where osteoarthritis (OA) was stated as an indication for surgery but, further, sequentially excluded any that reported, in addition to OA, fractured neck of femur (1074), fractured acetabulum (209) and previous arthrodesis or failed hemiarthroplasty (253), leaving 799,019 for initial analysis (Fig. 1), with a maximum potential follow-up of 13.75 years.

Surgical approach was grouped as (i) posterior, (ii) lateral, anterolateral, Hardinge (an eponymous name for a variant of the lateral approach), (iii) anterior or other and (iv) trans-trochanteric (overriding the others). These groups were further subdivided according to whether or not minimally invasive surgery (defined as skin incision lengths of less than 10 cm) was used, giving 8 potential subgroups. There was a secular increase in the use of the posterior approach (Additional file 1: Table S1 and Fig. S1). In total, 40,552 (5·1%) of the operations used minimally invasive surgery; the proportions of minimally invasive cases per year have decreased and plateaued (Additional file 1: Fig. S2).

Additional file 1: Table S2 summarises the characteristics of those in each approach group, including the type of implant used. As body mass index (BMI) was not collected in the early years of the registry (MDS v1) and data completeness for BMI improved over time, the data on this variable were not available for all patients. Metal-on-metal (MoM) hip replacements (including resurfacings) are strongly associated with higher failure rates [13] and have now largely fallen into disuse, so we focussed on the remaining 724,165 (90·6%) non-MoM hip replacements. Finally, as only 261 of these THRs underwent minimally invasive trans-trochanteric surgery, this group was excluded, leaving 723,904 for analysis (Fig. 1). Demographics for the final 7 groups are shown in Table 1.

**Table 1:** Numbers in the approach groups with demographic information

**Approach**	**Minimally invasive procedure used**	**Number (%)**	**Males**	Median (IQR) age at primary (years)*	**Other indication for primary (than just OA)**	**ASA**				**Fixation**			
						**P1**	**P2**	**P3**	**P4/P5**	**Cemented**	**Un-cemented**	**Hybrid**	**Reverse hybrid**
**Posterior**	**No**	394,735 (54.5)	39.2%	70 (62–76)	3.5%	15.0%	70.1%	14.5%	0.4%	32.3%	39.8%	24.5%	3.4%
**Posterior**	**Yes**	22,663 (3.1)	38.6%	69 (62–76)	2.8%	20.4%	70.0%	9.2%	0.3%	13.4%	72.9%	10.8%	3.0%
**Lat/Ant-Lat/Hard**	**No**	260,415 (36.0)	38.2%	71 (64–77)	2.6%	14.0%	70.0%	15.9%	0.6%	46.0%	36.6%	15.3%	2.1%
**Lat/Ant-Lat/Hard**	**Yes**	9,101 (1.3)	36.6%	71 (64–78)	2.4%	19.3%	68.1%	12.3%	0.3%	33.9%	51.8%	12.9%	1.4%
**Ant/Other**	**No**	26,582 (3.7)	38.1%	70 (63–77)	3.1%	13.4%	71.5%	14.7%	0.4%	32.6%	43.6%	22.0%	1.8%
**Ant/Other**	**Yes**	3,830 (0.5)	41.0%	69 (61–75)	3.1%	18.7%	66.7%	14.3%	0.3%	7.0%	83.7%	8.9%	0.5%
**Trans-trochanteric**	**No**	6,578 (0.9)	39.7%	69 (62–76)	5.2%	15.6%	69.0%	15.0%	0.3%	85.8%	8.1%	5.0%	1.1%
**Approach**	**Minimally invasive procedure used**	**Number (%)**	**Head size**			**BMI (kg/m**^**2**^**)**							
			**Number available**	**Large head (36 mm+)**		***% missing***	**Number available**		**Normal (19–25)**	**Under-weight (< 19)**	**Overweight (26–30)**	**Obese (> 30)**	
**Posterior**	**No**	394,735 (54.5)	394,671	25.5%		*33.4%*	262,876		27.9%	0.8%	39.5%	31.8%	
**Posterior**	**Yes**	22,663 (3.1)	22,662	36.8%		*32.2%*	15,371		35.4%	1.1%	40.3%	23.2%	
**Lat/Ant-Lat/Hard**	**No**	260,415 (36.0)	260,376	14.0%		*45.3%*	142,430		27.1%	0.8%	39.7%	32.4%	
**Lat/Ant-Lat/Hard**	**Yes**	9,101 (1.3)	9,097	12.1%		*54.1%*	4,175		32.1%	1.1%	40.6%	26.2%	
**Ant/Other**	**No**	26,582 (3.7)	26,579	22.0%		*43.4%*	15,047		28.0%	0.8%	40.0%	31.2%	
**Ant/Other**	**Yes**	3,830 (0.5)	3,829	41.0%		*49.0%*	1,952		30.4%	1.0%	44.5%	24.1%	
**Trans-trochanteric**	**No**	6,578 (0.9)	6,578	4.1%		*71.2%*	1,894		31.1%	1.3%	40.0%	27.6%	

*Lat/Ant-Lat/Hard* lateral/anterolateral/Hardinge, *Ant/Other* anterior/other, *IQR* interquartile range, *OA* osteoarthritis, *ASA* American Society of Anesthesiologists Physical Status, *BMI* body mass index

*Age regarded as missing for 157 as the NHS number was not validated

### Revision and short-term mortality

Survival analyses were used to compare the revision rates and 90-day mortality between groups. The unit of analysis was the THR. Although 5967 had been implanted as part of a bilateral pair, these were retained in both sets of analyses as they did not necessarily have the same approach. For revision, time was measured from the date of the primary to the date of first revision, if any, censoring at the end of 2016 or at death if that occurred first. For mortality, time was measured from the date of the primary to death, censoring at 90 days or at the end of 2016 if that was earlier.

Confounders for both sets of analyses were age at primary, sex, ‘risk group’ (i.e. had additional indications to OA for surgery), American Society of Anesthesiologists (ASA) grade and THR fixation. Year of primary, femoral head size and BMI (values < 10 and > 60 kg/m^2^ were excluded as likely to be invalid) were also considered.

For mortality, other known confounders were thromboprophylaxis and anaesthetic [3]. Also included was a measure of area deprivation, the Index of Multiple Deprivation (IMD), obtained via linkage to Hospital Episode Statistics (HES) inpatient records (for patients with National Health Service (NHS)-funded procedures in England). The IMD is an overall ranking of the patients’ ‘Super Output Area’ of residence, rank 1 being the most deprived area, 32,482 the least; we grouped the patients according to the quintile of their area rank.

A series of Cox ‘proportional hazards’ (PH) regression models were used to compare outcomes between groups, with stratification to allow subgroups of confounder variables to have different baseline hazard rates. For revision, the analyses were supplemented with Flexible Parametric Survival Modelling (FPM) [14]. The latter gave the analyses more scope and allowed exploration of the temporal changes in the effects of confounder variables as previously utilised in NJR analyses [13].

### Patient-reported outcome measures (PROMs)

The NHS PROM programme has collected PROMs for unilateral THRs in England since 1 April 2009 [15]. Patients complete validated PROM questionnaires 2 weeks before their primary operation (Q1) and approximately 6 months afterwards (Q2). Each questionnaire includes (a) the Oxford Hip Score (OHS) [16], derived from 12 questions asking about pain and mobility (over the previous 4 weeks); (b) the EuroQol-5D (EQ-5D) Health Scale [17], a visual analogue scale representing the patient’s health status on the day (0 = worst imaginable; 100 = best imaginable) and (c) the EQ-5D-3L Index (3 response options for mobility, self-care, performing usual activities, pain/discomfort and anxiety/depression). For (c), we looked at the responses to each of the 5 questions, rather than the composite ‘Index’. We also report on additional questions asked at Q2 about self-reported post-operative problems.

The NJR was linked to the PROM dataset via HES identifiers. PROM data were incomplete. Where multiple PROMs had been collected, the best ‘episode matched rank’ was used.

Statistical methods included regression analyses for OHS at Q2 with covariate adjustment for differences at Q1 (using fractional polynomials to linearise the relationship between Q2 and Q1), logistic regression (binary target variable) and ordered logistic (proportional odds) models for ordered categories. Non-parametric analyses (Kruskal-Wallis test) were used for complementary analyses where distributional assumptions were not met satisfactorily and followed by pairwise comparisons with Dunn’s method.

Throughout, the ‘conventional’ posterior approach (i.e. without minimally invasive surgery) group was used as the reference; further comparisons were made between minimally invasive surgery ‘yes’ vs. ‘no’ within other main approach groups. No adjustments for multiple comparisons have been made. All statistical analyses were conducted using Stata version 14·2 (Stata/SE 14·2 software, StataCorp LLC, Texas, 1985–2015).

## Results

### Revision

**Fig. 2:**
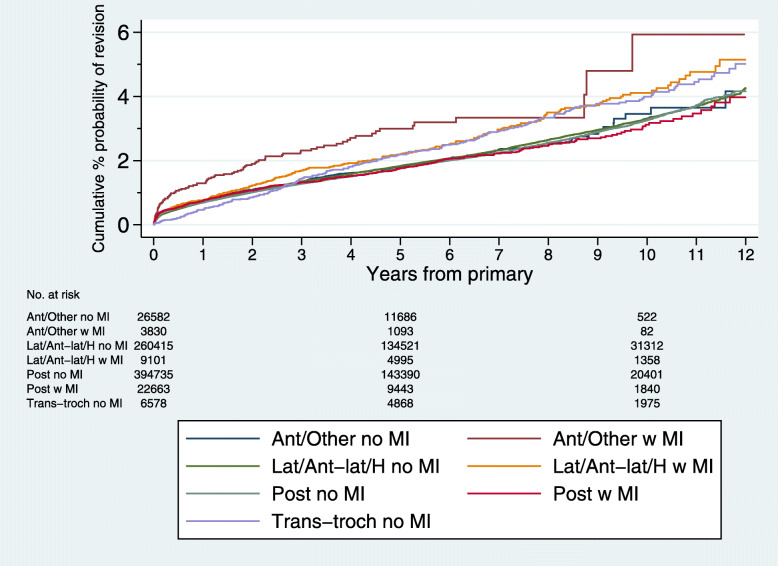
Cumulative percentage revised (Kaplan-Meier) up to 12 years for the 7 surgical approach groups

A total of 12,989 (1.8%) of 723,904 implants were revised during follow-up; 84,294 (11.6%) died without undergoing revision. Indications for revision surgery by primary surgery approach group are summarised (Additional file 1: Table S3). Figure 2 shows the estimated cumulative percentage revised (Kaplan-Meier) up to 12 years for the 7 approach groups.

There were some differences in possible confounding factors between groups (Table 1); we adjusted for these using two approaches. The first set of analyses used a Cox PH regression model stratifying by 16 age/sex/risk subgroups (age < 55, 55–64, 65–74 and 75+ years at primary; male vs. female; risk group, i.e. ‘other indication than just OA’, no vs. yes). Exploratory graphical checking suggested that the hazard rates for these subgroups were not proportional; hence, the stratification allowed their baseline hazards to differ. Table 2 (i) shows the results of these analyses. In total, 723,747 cases had complete data on age and sex and all had complete data on ASA and fixation. The middle column (Table 2 (i)) shows results with fixation and ASA as ‘fixed’ effects. Whilst both fixation and ASA were significant, graphical checking suggested their effects may be time-varying. However this was explored more using FPM described below. Year of primary was added to the model but was not statistically significant (*P* = 0.125, likelihood ratio test). Neither was femoral head size 36 mm and above in those where head size was known (*P* = 0.256; *n* = 723,635). Finally, BMI subgroup was added, as in the right-hand side of Table 2 (i) (*P* < 0.001). The unadjusted model showed that, compared with the conventional posterior approach, there was a higher risk of revision with the conventional lateral (*P* = 0.009), minimally invasive lateral (*P* < 0.001), minimally invasive anterior (*P* < 0.001) and trans-trochanteric (*P* = 0.004) approaches. Only the difference for the conventional lateral (*P* < 0.001) and trans-trochanteric (*P* = 0.003) approaches remained significant after adjustment.

**Table 2:** Regression models to compare approach groups for revision risk (*n* = 723,747 with complete information)

(i) Stratified Cox proportional hazards regression models, with stratification by age/sex/risk groups				
**Approach**	**Minimally invasive procedure used**	**Unadjusted Hazard rate ratio (95%CI)**	**With adjustment for fixation and ASA hazard rate ratio (95%CI)**	**With adjustment for fixation, ASA and BMI subgroup (*n*** **= 443,657) hazard rate ratio (95%CI)**
**Posterior**	**No**	1 [referent]	1 [referent]	1 [referent]
**Posterior**	**Yes**	0.99 [0.89–1.10] *P* = 0.864	0.92 [0.83–1.02] *P* = 0.110	0.89 [0.77–1.02] *P* = 0.097
**Lat/Ant-Lat/Hard**	**No**	1.05 [1.01–1.09] *P* = 0.009	1.07 [1.03–1.11] *P* = 0.001	1.12 [1.06–1.17] *P* < 0.001
**Lat/Ant-Lat/Hard**	**Yes**	1.31 [1.16–1.50] *P* < 0.001	1.28 [1.13–1.46] *P* < 0.001	1.02 [0.80–1.30] *P* = 0.861
**Ant/Other**	**No**	1.04 [0.95–1.14] *P* = 0.431	1.03 [0.94–1.13] *P* = 0.561	1.01 [0.88–1.15] *P* = 0.921
**Ant/Other**	**Yes**	1.67 [1.36–2.05] *P* < 0.001	1.48 [1.21–1.82] *P* < 0.001	1.03 [0.71–1.51] *P* = 0.870
**Trans-trochanteric**	**No**	1.22 [1.07–1.40] *P* = 0.004	1.40 [1.22–1.60] *P* < 0.001	1.48 [1.14–1.91] *P* = 0.003
***Additional pairwise comparisons:***				
*Lat/Ant-Lat/Hard*	*No* vs. *Yes*	*P* = 0.001	*P* = 0.005	*P* = 0.475
*Ant/Other*	*No* vs. *Yes*	*P* < 0.001	*P* = 0.001	*P* = 0.902
(ii) FPM models, with adjustment for time-varying effects of age, sex, risk group				
**Approach**	**Minimally invasive procedure used**	**Unadjusted Coefficient (95%CI)**	**With adjustment for fixation and ASA, as time-varying effects Coefficient (95%CI)**	
**Posterior**	**No**	0 [Referent]	0 [Referent]	
**Posterior**	**Yes**	−0.006 [−0.109–0.096] *P* = 0.903	−0.081 [−0.183–0.022] *P* = 0.125	
**Lat/Ant-Lat/Hard**	**No**	0.056 [0.019–0.093] *P* = 0.003	0.069 [0.031–0.106] *P* < 0.001	
**Lat/Ant-Lat/Hard**	**Yes**	0.282 [0.154–0.411] *P* < 0.001	0.264 [0.135–0.392] *P* < 0.001	
**Ant/Other**	**No**	0.031 [−0.063–0.126] *P* = 0.516	0.019 [−0.075–0.114] *P* = 0.688	
**Ant/Other**	**Yes**	0.516 [0.311–0.721] *P* < 0.001	0.380 [0.174–0.585] *P* < 0.001	
**Trans-trochanteric**	**No**	0.213 [0.075–0.350] *P* = 0.002	0.309 [0.170–0.448] *P* < 0.001	
***Additional pairwise comparisons:***				
*Lat/Ant-Lat/Hard*	*No* vs. *Yes*	*P* = 0.001	*P* = 0.003	
*Ant/Other*	*No* vs. *Yes*	*P* < 0.001	*P* = 0.002	

*Lat/Ant-Lat/Hard* lateral/anterolateral/Hardinge, *Ant/Other* anterior/other, *ASA* American Society of Anesthesiologists Physical Status, *BMI* body mass index, *CI* confidence interval

The second set of analyses used FPM. The baseline hazard was modelled with 4 degrees of freedom (df); time-varying effects of age (as 4 restricted cubic splines), sex and risk group were modelled with 4, 1 and 1 df respectively (Table 2 (ii)). Further adjustment was made for fixation and ASA (here after combining P3 and P4/P5) which were also time-varying (and modelled with df = 2 and 3 respectively). We were unable to obtain convergence when we added BMI to this model. Similar to the Cox model, the unadjusted analyses showed a higher risk of revision for the conventional lateral (*P* = 0.003), minimally invasive lateral (*P* < 0.001), minimally invasive anterior (*P* < 0.001) and trans-trochanteric (*P* = 0.002) approaches. These findings persisted in the adjusted models (conventional lateral (*P* < 0.001), minimally invasive lateral (*P* < 0.001), minimally invasive anterior (*P* < 0.001) and trans-trochanteric (*P* < 0.001)).

The FPM model demonstrated that the minimally invasive lateral and anterior approaches have higher failure rates than their corresponding conventional approaches, but with the Cox model this effect dissipates with adjustment for BMI (Table 2 (i)).

### PROMs

PROMs were able to be linked to 289,296 (54%) of the 533,477 unilateral THRs undertaken since PROM monitoring began. The cohort with PROMs was broadly similar to the remainder over this period in respect of age, sex, ASA, risk group and fixation (data not shown but available).

#### Oxford Hip Scores (OHS)

The pre-operative measurements (Q1) were normally distributed, but the post-operative measurements (Q2) were highly skewed (Additional file 1: Fig. S3) with a median score of 42 (IQR 35–46). There were differences between the groups in pre-operative Q1 scores (Additional file 1: Table S4).

Additional file 1: Table S5 shows between group comparisons of the Q2 scores. A non-parametric comparison (i) is shown because of extreme skewness. To adjust for differences in Q1, a regression model was sought. The relationship between Q2 and Q1, however, was non-linear; fractional polynomials of Q1 were explored in conjunction with the regression and the transformation that best linearised this relationship was ln(1 + Q1 score). Comparisons between the mean Q2 scores with adjustment for differences in (transformed) Q1 scores are shown in (ii). The results were virtually unchanged from (i) except the difference between (3) and (4) (effect of minimally invasive surgery for ‘Lat/Ant-Lat/Hard’) was no longer significant. Residuals from this analysis were only approximately normal, but analysis using robust variance estimation (shown in parenthesis) left the results unchanged.

Additional file 1: Table S5 also shows the improvements in OHS between Q1 and Q2, calculated simply from ‘Q2 minus Q1’. Because of the ceiling effect of the OHS (the maximum attainable score being 48), the potential to ‘improve between Q1 and Q2’ depends on the initial score; the right-hand side column of Additional file 1: Table S5 shows the ‘potential’ for improvement, calculated from ‘48 minus Q1’.

Both of these showed statistically, but not clinically [16], significant differences between the groups. We also repeated (i) and (ii) using proportional odds ordered logistic regression (not shown) with similar findings.

#### EQ-5D-3L Health Index

Analysis of the EQ-5D Health Scale (Visual Analogue Score) results also showed statistically, but not clinically significant differences between the subgroups (Additional file 1: Table S6).

EQ-5D-3L Health Index question responses are shown for complete pairs (Q1 and Q2) in Additional file 1: Tables S7 (a) to (e). Responses to each question took the form of 3 ordered categories as listed in these tables. In each case, logistic regression analyses were used to compare the approach groups at Q2, after first combining 2 of the 3 categories as indicated in the footnote of the table; both adjusted and unadjusted models showed differences between the approaches.

#### Self-reported post-operative problems

We analysed the PROM self-reported post-operative problems (Additional file 1: Table S8). There were significant differences between groups. Comparing the 2 commonest approaches, the lateral conventional approach was associated with slightly higher rates of complications than the conventional posterior approach.

### 90-day mortality

**Fig. 3:**
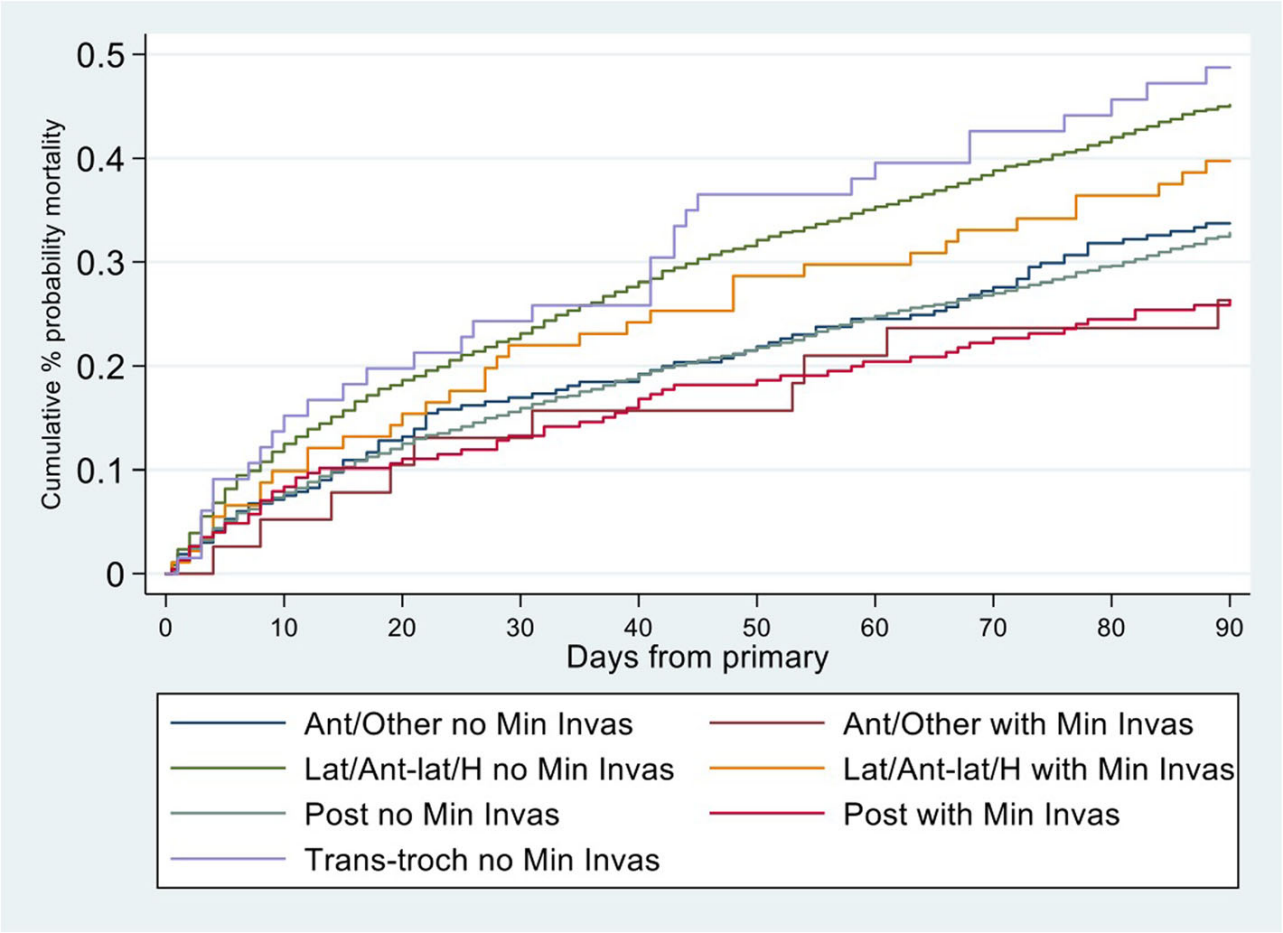
Cumulative mortality up to 90 days (Kaplan-Meier) for the 7 surgical approach groups

Figure 3 shows the cumulative mortality up to 90 days (Kaplan-Meier) for the 7 groups (logrank *P* < 0.001).

Our previous work on mortality after hip replacement [3] had identified confounding factors, and a series of univariable analyses (data not shown, but available on request) confirmed these. Thus, in our analysis shown in Table 3, we have adjusted for these factors. Analyses included a stratification for ‘risk group’, as the hazard rates were not proportional over time. Age and year of primary THR were analysed as continuous variables, rather than ‘grouped’, and modelled using restricted cubic splines. In all models, the conventional lateral approach was associated with a higher risk of mortality than the conventional posterior approach. There were no other significant differences in mortality compared to the referent conventional posterior approach group. Similar findings were demonstrated when models were also adjusted for Charlson comorbidity subgroups, which were based on HES inpatient admissions within 5 years prior to date of primary THR operation (Table 4).

**Table 3:** Cox ‘proportional hazards’ regression model to compare 90-day mortality between the 7 approach subgroups

**Approach**	**Minimally invasive procedure used**	**Number for analysis**	**(i) Unadjusted model (*n*** **= 723,747; 2673 deaths) hazard rate ratio [95%CI]**	**(ii) With covariate adjustment for sex, age, ASA and year of primary, stratified by ‘risk group’ (*n*** **= 723,747; 2673 deaths) hazard rate ratio [95%CI]**	**(iii) With covariate adjustment for sex, age, ASA, year of primary and fixation, stratified by ‘risk group’ (*n*** **= 723,747; 2673 deaths) hazard rate ratio [95%CI]**
**Posterior**	**No**	394,655	1 [referent]	1 [referent]	1 [referent]
**Posterior**	**Yes**	22,655	0.80 [0.62–1.04] *P* = 0.101	0.88 [0.68–1.14] *P* = 0.344	0.90 [0.69–1.17] *P* = 0.447
**Lat/Ant-Lat/Hard**	**No**	260,353	1.38 [1.27–1.49] P < 0.001	1.14 [1.05–1.24] P = 0.002	1.14 [1.05–1.23] P = 0.002
**Lat/Ant-Lat/Hard**	**Yes**	9099	1.21 [0.87–1.69] *P* = 0.252	1.02 [0.73–1.43] *P* = 0.896	1.03 [0.74–1.44] *P* = 0.847
**Ant/Other**	**No**	26,578	1.03 [0.83–1.28] *P* = 0.793	0.96 [0.77–1.19] *P* = 0.703	0.96 [0.77–1.19] *P* = 0.715
**Ant/Other**	**Yes**	3829	0.80 [0.43–1.50] *P* = 0.493	0.88 [0.47–1.65] *P* = 0.697	0.91 [0.49–1.71] *P* = 0.780
**Trans-trochanteric**	**No**	6578	1.49 [1.05–2.11] P = 0.026	1.19 [0.83–1.69] *P* = 0.342	1.16 [0.81–1.65] *P* = 0.412
*Additional pairwise comparisons:*					
*Lat/Ant-Lat/Hard*	*No* vs. *Yes*		*P = 0.452*	*P = 0.517*	*P = 0.573*
*Ant/Other*	*No* vs. *Yes*		*P = 0.460*	*P = 0.807*	*P = 0.884*
**Approach**	**Minimally invasive procedure used**	**(iv) With covariate adjustment for sex, age, ASA, year of primary, fixation, mechanical and chemical thromboprophylaxis and anaesthetic group, stratified by ‘risk group’ (*n*** **= 713,994 with complete information; 2621 deaths) hazard rate ratio [95%CI]**		**(v) With covariate adjustment for sex, age, ASA, year of primary, fixation, mechanical and chemical thromboprophylaxis, anaesthetic group and quintile of area deprivation, stratified by ‘risk group’ (*n*** **= 572,719 with complete information; 2192 deaths) hazard rate ratio [95%CI]**	**(vi) With covariate adjustment for sex, age, ASA, year of primary, fixation, mechanical and chemical thromboprophylaxis, anaesthetic group, quintile of area deprivation and BMI subgroup, stratified by ‘risk group’ (*n*** **= 359,883 with complete information; 1148 deaths) hazard rate ratio [95%CI]**
**Posterior**	**No**	1 [referent]		1 [referent]	1 [referent]
**Posterior**	**Yes**	0.91 [0.70–1.18] *P* = 0.475		0.93 [0.68–1.27] *P* = 0.644	0.87 [0.57–1.34] *P* = 0.525
**Lat/Ant-Lat/Hard**	**No**	1.16 [1.06–1.26] P = 0.001		1.11 [1.01–1.21] P = 0.027	1.15 [1.01–1.30] P = 0.029
**Lat/Ant-Lat/Hard**	**Yes**	0.91 [0.63–1.31] *P* = 0.605		0.87 [0.58–1.31] *P* = 0.512	0.80 [0.41–1.55] *P* = 0.507
**Ant/Other**	**No**	0.96 [0.78–1.20] *P* = 0.732		0.94 [0.75–1.19] *P* = 0.620	0.89 [0.63–1.26] *P* = 0.509
**Ant/Other**	**Yes**	0.92 [0.49–1.71] *P* = 0.787		1.23 [0.61–2.47] *P* = 0.563	1.01 [0.32–3.15] *P* = 0.986
**Trans-trochanteric**	**No**	1.16 [0.81–1.67] *P* = 0.409		1.12 [0.75–1.68] *P* = 0.571	1.12 [0.52–2.41] *P* = 0.771
*Additional pairwise comparisons:*					
*Lat/Ant-Lat/Hard*	*No* vs *Yes*	*P = 0.195*		*P = 0.250*	*P = 0.283*
*Ant/Other*	*No* vs *Yes*	*P = 0.885*		*P = 0.447*	*P = 0.833*

*Lat/Ant-Lat/Hard* lateral/anterolateral/Hardinge, *Ant/Other* anterior/other, *ASA* American Society of Anesthesiologists Physical Status, *BMI* body mass index, *CI* confidence interval

**Table 4:** Cox ‘proportional hazards’ regression model to compare 90-day mortality between the 7 approach subgroups. Models from Table 3 were also adjusted for Charlson comorbidity subgroups, which were based on HES inpatient admissions within 5 years prior to date of primary THR operation

**Approach**	**Minimally invasive procedure used**	**(i) With covariate adjustment for sex, age, ASA, year of primary, fixation, mechanical and chemical thromboprophylaxis, anaesthetic group and Charlson comorbidity subgroups, stratified by ‘risk group’ (*n*** **= 578,624 with complete information; 2205 deaths) hazard rate ratio [95%CI]**	**(ii) With covariate adjustment for sex, age, ASA, year of primary, fixation, mechanical and chemical thromboprophylaxis, anaesthetic group, Charlson comorbidity subgroups and quintile of area deprivation, stratified by ‘risk group’ (*n*** **= 572,719 with complete information; 2192 deaths) hazard rate ratio [95%CI]**	**(iii) With covariate adjustment for sex, age, ASA, year of primary, fixation, mechanical and chemical thromboprophylaxis, anaesthetic group, Charlson comorbidity subgroups, quintile of area deprivation and BMI subgroup, stratified by ‘risk group’ (*n*** **= 359,883 with complete information; 1148 deaths) hazard rate ratio [95%CI]**
**Posterior**	**No**	1 [referent]	1 [referent]	1 [referent]
**Posterior**	**Yes**	0.94 [0.69–1.28] *P* = 0.705	0.94 [0.69–1.28] *P* = 0.678	0.86 [0.56–1.33] *P* = 0.499
**Lat/Ant-Lat/Hard**	**No**	1.13 [1.04–1.24] P = 0.007	1.12 [1.02–1.23] P = 0.013	1.16 [1.02–1.31] P = 0.022
**Lat/Ant-Lat/Hard**	**Yes**	0.89 [0.59–1.34] *P* = 0.579	0.88 [0.59–1.33] *P* = 0.555	0.81 [0.42–1.57] *P* = 0.539
**Ant/Other**	**No**	0.96 [0.76–1.21] *P* = 0.726	0.96 [0.76–1.21] *P* = 0.706	0.90 [0.64–1.27] *P* = 0.549
**Ant/Other**	**Yes**	1.29 [0.64–2.59] *P* = 0.473	1.32 [0.66–2.65] *P* = 0.435	1.10 [0.35–3.42] *P* = 0.872
**Trans-trochanteric**	**No**	1.15 [0.77–1.73] *P* = 0.486	1.15 [0.77–1.71] *P* = 0.506	1.16 [0.54–2.50] *P* = 0.701
*Additional pairwise comparisons:*				
*Lat/Ant-Lat/Hard*	*No* vs *Yes*	*P = 0.247*	*P = 0.253*	*P = 0.296*
*Ant/Other*	*No* vs *Yes*	*P = 0.425*	*P = 0.386*	*P = 0.741*

*Lat/Ant-Lat/Hard* lateral/anterolateral/Hardinge, *Ant/Other* anterior/other, *ASA* American Society of Anesthesiologists Physical Status, *BMI* body mass index, *CI* confidence interval

*There were no 90-day deaths for human immunodeficiency virus so this was not included in the analysis

Duplication of data by inclusion of the bilateral implants did not impact the findings; only 17 of the 2675 90-day deaths were associated with bilateral THRs.

## Discussion

We have compared the 7 common surgical approaches to the hip joint used in THR by risk of revision, PROMs and 90-day mortality. In the fully adjusted Cox model, the conventional lateral and conventional trans-trochanteric approaches were associated with higher risks of revision compared to the conventional posterior approach. All other approaches (including all minimally invasive approaches) were not associated with an increased revision risk. However, the fully adjusted FPM which accounts for time-varying effects showed higher risk of revision with both the conventional and minimally invasive lateral approaches and the minimally invasive anterior and the trans-trochanteric approaches.

Composite PROM scores showed statistical, but not clinically significant, differences. This is similar to findings from other registries [18]. Self-reported specific complications such as bleeding and reoperation were higher with the conventional lateral approach than the conventional posterior approach, but again, there were no other consistent meaningful differences found. Ninety-day mortality was higher in the conventional lateral approach group when compared with the conventional posterior approach, but there were no other significant differences.

Registry data is observational and thus causation cannot be attributed. However, the associations shown here are consistent even with thorough adjustment for confounders. Some BMI data is missing as this was not collected in the early years of the NJR. Previously when analysing this dataset, we have used multiple imputation for BMI data, but this did not affect the results [3], suggesting data was missing at random. Any missing revisions would also be expected to be missing at random when analysing by primary approach. Registry data is generalisable as it encompasses the entire population and is thus less susceptible to selection and reporting bias. Some iterations of the NJR MDS and reporting of the data from those versions of the MDS did not specify ‘anterior’ as a separate category of approach, but included it with ‘other’; therefore, these groups were combined. It is possible that some of the cases defined as ‘other’ were not anterior and did not fit into any of the other approach groups defined in this analysis or their minimally invasive variants, but we believe the number of such cases would be small given the limited options for other hip approaches.

Furthermore, limited information is collected in the NJR regarding what specific minimally invasive surgical techniques were used, other than the use of a small skin incision, with these approaches only accounting for a small subgroup (5.1%) of the cohort. Although we recognise further research is needed into defining and categorising minimally invasive approaches, we considered it important to include these patients given a conscious effort was made by the surgeon to minimise the incision length, which may influence the technical performance and/or outcome of the THR.

A number of small randomised controlled trials (RCTs) have compared the effect of two THR approaches; however, these have limitations. Cheng et al. (*n* = 72) observed that the anterior approach had similar results at 12 weeks to the posterior approach in terms of complications, patient-reported outcomes and gait, aside from neuropraxia which was more common following the anterior approach [11]. Taunton et al. (*n* = 101) found that although there were some early benefits of the anterior approach (e.g. earlier discontinuation of walking aids), it was similar to the minimally invasive posterior approach at 1 year for PROMs, complications and radiographic parameters [19]. Two trials comparing the lateral with the posterior approach showed the posterior approach had greater improvement in muscle strength at 1 year (*n* = 47) [20], and fewer dislocations at an average 3-year follow-up (*n* = 196) [21]. A recent RCT (*n* = 164) found the lateral approach was associated with more abductor muscle weakness and worse PROMs, but the anterior approach had more nerve injuries [22]. A systematic review, including 12 RCTs, reported similar risks of dislocation, nerve injury, infection and venous thromboembolism between the posterior approach and the minimally invasive posterior approach [10].

The RCTs comparing surgical approach are not large enough to compare less common but important events such as revision and mortality rates. We observed that the conventional lateral approach was associated with higher revision and mortality rates than the conventional posterior approach. Previous studies have also observed higher revision rates (for all causes apart from dislocation) [23] and higher mortality rates [3] with the lateral compared to the posterior approach. The posterior approach is a more muscle-sparing approach compared with the lateral approach and is associated with less bleeding [8]. Problems seen more commonly following the anterolateral approach include nerve injury [8], reduced muscle strength [9] and limping [10]. These problems invariably influence patient mobility, especially in the early post-operative phase, when the mortality risk is higher. Previous work on mortality after THR showed lower all-cause mortality, and mortality from respiratory issues with the posterior approach; this finding is most likely related to improved mobility after surgery [24]. It is therefore plausible that a combination of these factors contribute to the higher revision and mortality rates observed for the lateral approach compared to the posterior approach.

The conventional lateral approach (36.0%) is the second most popular approach and is currently used annually in over 20,000 primary THRs in the NJR. This approach was associated with worse outcomes in all measures than the commonest approach, the conventional posterior (54.5%). Therefore, the data presented here does not support its continued use over alternative approaches. Although the conventional trans-trochanteric approach was associated with higher revision rates (compared with the posterior approach), it is acknowledged that this approach is rarely used now, and when it is used it is usually for more complex cases (such as hip ankylosis, proximal femoral deformities and acetabular protrusio), which may explain the inferior outcomes observed here with the conventional trans-trochanteric approach. By contrast, the posterior approach is common, extensile and associated with good outcomes in comparison to other approaches. Therefore, we recommend the posterior approach should be considered the current standard surgical approach for THR. It would be difficult and perhaps unwise to attempt conversion of experienced surgeons to an approach with which they may be unfamiliar. However, surgeons in training should be taught alternative approaches to the lateral associated with better outcomes, like the posterior approach. The data does support continued use of minimally invasive approaches, with acceptable mortality and PROM outcomes, although minimally invasive lateral and anterior approaches may be associated with higher revision rates than their corresponding conventional approaches.

## Conclusion

The lateral approach was associated with worse outcomes, including more deaths and more revisions, than the posterior approach for primary THR. We thus recommend that new surgeons do not routinely use the lateral approach for THR. The conventional posterior approach is common and associated with at least as good outcomes as other less common approaches; therefore, we recommend the posterior approach should be considered the current standard approach for THR and should be used when training future surgeons. As minimally invasive posterior approaches and the conventional anterior approach had outcomes in our observational data similar to the conventional posterior approach in all domains, these should be assessed further in randomised controlled trials and compared with the conventional posterior approach.

## Supplementary information

**Additional file 1.** Summary information including temporal trends of clinical practice, detailed demographics of the cohort divided by surgical approach, indications for revision surgery, and detailed analyses of patient reported outcome measures. **Fig. S1.** Numbers of primaries using each surgical approach in each year. **Fig. S2.** (a) Numbers of primaries using minimally invasive surgery (No vs. Yes), by year of primary, (b) Percentage of primaries using minimally invasive surgery, by year of primary. **Fig. S3.** Distribution of post-operative (Q2) OHS available for 237,660 hips, with a Normal distribution superimposed (OHS score range 0 to 48 with 48 being the best possible score). **Table S1.** Surgical approach by year of primary. **Table S2.** Numbers in the approach subgroups together with some demographic information. **Table S3.** Indications for revision surgery by surgical approach used at the primary procedure. **Table S4.** Comparison of pre-operative (Q1) PROMS Oxford Hip Scores (OHS) between the approach subgroups. **Table S5.** Comparison of PROMS OHS scores between the approach subgroups. **Table S6.** Comparison of the EQ-5D Health Scale (Visual Analogue Score) between the approach subgroups. **Table S7.** Comparison between the approach subgroups in respect of the responses to the 5 component questions of the PROMs EQ-5D Health Index: (a) Mobility, (b) Self care, (c) Usual activity, (d) Pain/discomfort, (e) Anxiety/depression. **Table S8.** Other PROMS - comparison of patient self-report post-operative (Q2) problems between the approach subgroups.

## Data Availability

Access to data is available from the National Joint Registry for England, Wales, Northern Ireland and the Isle of Man, but restrictions apply to the availability of these data, which were used under licence for the current study, and so are not publicly available. Data access applications can be made to the National Joint Registry Research Committee. Access to linked HES and PROM data is available through data applications to NHS Digital.

## Abbreviations

ASA: American Society of Anesthesiologists
BMI: Body mass index
CI: Confidence intervals
df: Degrees of freedom
EQ-5D: EuroQol-5D
FPM: Flexible Parametric Survival Modelling
HRR: Hazard rate ratio
HES: Hospital Episode Statistics
IMD: Index of Multiple Deprivation
IQR: Interquartile range
MoM: Metal-on-metal
MDS: Minimum data set
NHS: National Health Service
NJR: National Joint Registry
OA: Osteoarthritis
OHS: Oxford Hip Score
PROM: Patient-reported outcome measure
PH: Proportional hazards
RCT: Randomised controlled trial
THR: Total hip replacement
