# Supplementary material for: The effect of surgical approach in total hip replacement on outcomes: an analysis of 723,904 elective operations from the National Joint Registry for England, Wales, Northern Ireland and the Isle of Man
Source: BMC Med. 2020 Aug 6;18:242. doi: 10.1186/s12916-020-01672-0 (PMC7409663; doi:10.1186/s12916-020-01672-0)
Supplement: Supplementary file 1 — Additional file 1. Summary information including temporal trends of clinical practice, detailed demographics of the cohort divided by surgical approach, indications for revision surgery, and detailed analyses of patient reported outcome measures. Fig. S1. Numbers of primaries using each surgical approach in each year. Fig. S2. (a) Numbers of primaries using minimally invasive surgery (No vs. Yes), by year of primary, (b) Percentage of primaries using minimally invasive surgery, by year of primary. Fig. S3. Distribution of post-operative (Q2) OHS available for 237,660 hips, with a Normal distribution superimposed (OHS score range 0 to 48 with 48 being the best possible score). Table S1. Surgical approach by year of primary. Table S2. Numbers in the approach subgroups together with some demographic information. Table S3. Indications for revision surgery by surgical approach used at the primary procedure. Table S4. Comparison of pre-operative (Q1) PROMS Oxford Hip Scores (OHS) between the approach subgroups. Table S5. Comparison of PROMS OHS scores between the approach subgroups. Table S6. Comparison of the EQ-5D Health Scale (Visual Analogue Score) between the approach subgroups. Table S7. Comparison between the approach subgroups in respect of the responses to the 5 component questions of the PROMs EQ-5D Health Index: (a) Mobility, (b) Self care, (c) Usual activity, (d) Pain/discomfort, (e) Anxiety/depression. Table S8. Other PROMS - comparison of patient self-report post-operative (Q2) problems between the approach subgroups. [file 12916_2020_1672_MOESM1_ESM.docx]

**Additional file 1**

**Figure S1**

**Figure S2**

1. **Numbers of primaries using minimally invasive surgery (No vs. Yes), by year of primary**

1. **Percentage of primaries using minimally invasive surgery, by year of primary**

**Figure S3 Distribution of post-operative (Q2) OHS available for 237,660 hips, with a Normal distribution superimposed (OHS score range 0 to 48 with 48 being the best possible score).**

**Table S1 Surgical approach by year of primary**

| **Year of primary** | **Surgical approach:** | | | | **Total** |
| --- | --- | --- | --- | --- | --- |
|  | **Posterior** | **Antero-lateral, Hardinge or Lateral** | **Anterior or Other** | **Trans-trochanteric** |  |
| **2003** | 52 (44·1%) | 61 (51·7%) | 5 (4·2%) | 0 (0·0%) | **118 (100%)** |
| **2004** | 7,514 (38·6%) | 10,910 (56·0%) | 235 (1·2%) | 835 (4·3%) | **19,494 (100%)** |
| **2005** | 16,136 (42·2%) | 20,326 (53·1%) | 420 (1·1%) | 1,375 (3·6%) | **38,257 (100%)** |
| **2006** | 21,326 (46·9%) | 22,607 (49·7%) | 389 (0·9%) | 1,182 (2·6%) | **45,504 (100%)** |
| **2007** | 27,429 (48·2%) | 27,544 (48·4%) | 724 (1·3%) | 1,202 (2·1%) | **56,899 (100%)** |
| **2008** | 32,985 (53·1%) | 24,766 (39·8%) | 3,701 (6·0%) | 721 (1·2%) | **62,173 (100%)** |
| **2009** | 34,931 (55·2%) | 23,850 (37·7%) | 3,958 (6·3%) | 520 (0·8%) | **63,259 (100%)** |
| **2010** | 37,150 (56·8%) | 24,403 (37·3%) | 3,415 (5·2%) | 463 (0·7%) | **65,431 (100%)** |
| **2011** | 39,981 (58·8%) | 24,077 (35·4%) | 3,622 (5·3%) | 373 (0·6%) | **68,053 (100%)** |
| **2012** | 43,629 (61·2%) | 23,720 (33·3%) | 3,633 (5·1%) | 313 (0·4%) | **71,295 (100%)** |
| **2013** | 46,644 (64·2%) | 22,445 (30·9%) | 3,353 (4·6%) | 211 (0·3%) | **72,653 (100%)** |
| **2014** | 51,759 (65·8%) | 23,295 (29·6%) | 3,416 (4·3%) | 214 (0·3%) | **78,684 (100%)** |
| **2015** | 53,147 (68·0%) | 21,777 (27·9%) | 3,025 (3·9%) | 232 (0·3%) | **78,181 (100%)** |
| **2016** | 55,684 (70·5%) | 20,432 (25·9%) | 2,742 (3·5%) | 160 (0·2%) | **79,018 (100%)** |
| **All years** | **468,367 (58·6%)** | **290,213 (36·3%)** | **32,638 (4·1%)** | **7,801 (1·0%)** | **799,019 (100%)** |

**Table S2 Numbers in the approach subgroups together with some demographic information.**

| **Approach** | **Minimally invasive procedure used** | **Number** | **Males*** | **Median (IQR) age at primary** (years)** | **Other indication for primary (than just OA)** | **ASA** | | | | **Type of implant** | | | |
| --- | --- | --- | --- | --- | --- | --- | --- | --- | --- | --- | --- | --- | --- |
|  |  |  |  |  |  | **P1** | **P2** | **P3** | **P4/P5** | **Stemmed non-MoM** | **Stemmed MoM** | **Resurfacing** | **Uncertain** |
| **Posterior** | **No** | **442,257** | 41·6% | 69 (61-76) | 3·6% | 17·4% | 68·6% | 13·6% | 0·4% | 89·3% | 3·5% | 6·1% | 1·2% |
| **Posterior** | **Yes** | **26,110** | 40·2% | 68 (61-75) | 2·8% | 22·8% | 67·8% | 9·0% | 0·3% | 86·8% | 8·8% | 3·2% | 1·2% |
| **Lat/Ant-Lat/Hard** | **No** | **280,053** | 39·4% | 70 (63-77) | 2·6% | 15·0% | 69·0% | 15·5% | 0·5% | 93·0% | 3·4% | 2·3% | 1·3% |
| **Lat/Ant-Lat/Hard** | **Yes** | **10,160** | 37·9% | 70 (63-77) | 2·5% | 21·8% | 66·3% | 11·6% | 0·3% | 89·6% | 6·3% | 2·6% | 1·6% |
| **Ant/Other** | **No** | **28,650** | 39·2% | 70 (63-77) | 3·5% | 14·4% | 70·9% | 14·3% | 0·4% | 92·8% | 3·7% | 1·9% | 1·7% |
| **Ant/Other** | **Yes** | **3,988** | 41·3% | 69 (61-75) | 3·1% | 18·7% | 66·8% | 14·1% | 0·4% | 96·0% | 2·2% | 0·4% | 1·5% |
| **Trans-trochanteric** | **No** | **7,507** | 42·5% | 68 (60-75) | 5·0% | 18·2% | 67·6% | 13·9% | 0·3% | 87·6% | 3·8% | 6·9% | 1·7% |
| **Trans-trochanteric** | **Yes** | **294** | 41·5% | 69 (62-76) | 0·7% | 27·9% | 57·8% | 13·6% | 0·7% | 88·8% | 2·4% | 5·4% | 3·4% |

*1 had uncertain sex; **171 had missing/uncertain ages (170 couldn’t be validated; 1 was <0);

**(continued)**

| **Approach** | **Minimally invasive procedure used** | **BMI (kg/m^2^)** | | | | |  |
| --- | --- | --- | --- | --- | --- | --- | --- |
|  |  | **Number available** | **Normal**  **(19-25)** | **Underweight**  **(<19)** | **Overweight**  **(26-30)** | **Obese**  **(>30)** | |
| **Posterior** | **No** | **282,147** | 27·9% | 0·8% | 39·7% | 31·6% | |
| **Posterior** | **Yes** | **16,505** | 35·3% | 1·1% | 40·3% | 23·3% | |
| **Lat/Ant-Lat/Hard** | **No** | **149,066** | 27·0% | 0·8% | 39·8% | 32·4% | |
| **Lat/Ant-Lat/Hard** | **Yes** | **4,431** | 32·1% | 1·1% | 40·4% | 26·3% | |
| **Ant/Other** | **No** | **16,040** | 27·9% | 0·8% | 40·1% | 31·2% | |
| **Ant/Other** | **Yes** | **1,999** | 30·6% | 1·0% | 44·3% | 24·2% | |
| **Trans-trochanteric** | **No** | **2,085** | 30·9% | 1·3% | 39·9% | 27·9% | |
| **Trans-trochanteric** | **Yes** | **120** | 39·2% | 0·0% | 35·0% | 25·8% | |

**Table S3 Indications for revision surgery by surgical approach used at the primary procedure**

| **Approach** | **Minimally invasive procedure used** | **Number** | **Number**  **revised** | **Numbers (%) of revisions reporting each indication*:** | | | | | | | | | | | |
| --- | --- | --- | --- | --- | --- | --- | --- | --- | --- | --- | --- | --- | --- | --- | --- |
|  |  |  |  | **Aseptic loosening** | **Dislocation**  **/subluxation** | **Infection** | **Peri-prosthetic**  **fracture** | **Pain** | **Mal-**  **alignment** | **Implant**  **wear** | **Lysis** | **Implant**  **fracture** | **Head/**  **Socket**  **size mismatch** | **Other**  **indication** | **Adverse**  **soft**  **tissue**  **reaction**** |
| **Posterior** | **No** | 394,735 | **6,337** | 1,350  (21.3%) | 1,767  (27.9%) | 1,052  (16.6%) | 1,159 (18.3%) | 759  (12.0%) | 456  (7.2%) | 351  (5.5%) | 199  (3.1%) | 238  (3.8%) | 53  (0.8%) | 430  (6.8%) | 150/5,989  (2.5%) |
| **Posterior** | **Yes** | 22,663 | **390** | 95  (24.4%) | 93  (23.9%) | 40 (10.3%) | 71 (18.2%) | 42  (10.8%) | 40  (10.3%) | 27  (6.9%) | 11  (2.8%) | 19  (4.9%) | 7  (1.8%) | 29  (7.4%) | 15/357  (4.2%) |
| **Lat/Ant-Lat/Hard** | **No** | 260,415 | **5,254** | 1,649 (31.4%) | 971  (18.5%) | 1,059 (20.2%) | 734 (14.0%) | 713  (13.6%) | 526  (10.0%) | 391  (7.4%) | 283  (5.4%) | 202  (3.8%) | 49  (0.9%) | 343  (6.5%) | 129/4,763  (2.7%) |
| **Lat/Ant-Lat/Hard** | **Yes** | 9,101 | **242** | 83  (34.3%) | 40  (16.5%) | 41 (16.9%) | 36 (14.9%) | 35  (14.5%) | 24  (9.9%) | 21  (8.7%) | 18  (7.4%) | 6  (2.5%) | 2  (0.8%) | 23  (9.5%) | 0/195  (0.0%) |
| **Ant/Other** | **No** | 26,582 | **461** | 145  (31.5%) | 77  (16.7%) | 97 (21.0%) | 70 (15.2%) | 67  (14.5%) | 46  (10.0%) | 29  (6.3%) | 22  (4.8%) | 15  (3.3%) | 4  (0.9%) | 35  (7.6%) | 15/452  (3.3%) |
| **Ant/Other** | **Yes** | 3,830 | **93** | 25  (26.9%) | 6  (6.5%) | 19  (20.4%) | 22 (23.7%) | 11  (11.8%) | 8  (8.6%) | 9  (9.7%) | 1  (1.1%) | 2  (2.2%) | 3  (3.2%) | 5  (5.4%) | 3/85  (3.5%) |
| **Troch Osteotomy** | **No** | 6,578 | **212** | 70  (33.0%) | 58  (27.4%) | 51  (24.1%) | 13  (6.1%) | 37  (17.5%) | 14  (6.6%) | 10  (4.7%) | 14  (6.6%) | 9  (4.3%) | 0  (0.0%) | 9  (4.3%) | 0/183  (0.0%) |
| **Total** | | **723,904** | **12,989** | **3.417 (26.3%)** | **3,012**  **(23.2%)** | **2,359 (18.2%)** | **2,105**  **(16.2%)** | **1,664**  **(12.8%)** | **1,114**  **(8.6%)** | **838**  **(6.5%)** | **548 (4.2%)** | **491**  **(3.8%)** | **118**  **(0.9%)** | **874**  **(6.7%)** | **312/12,024**  **(2.6%)** |

**The listed indications are not mutually exclusive; any number of indications may be recorded for each hip revision*

***This indication was not used in the registry’s early data collection forms (ie pre MDS vs 3).*

**Table S4 Comparison of pre-operative (Q1) PROMS Oxford Hip Scores (OHS) between the approach subgroups.**

| **Approach** | **Minimally invasive procedure used** | **All available**  **pre-operative OHS** | |
| --- | --- | --- | --- |
|  |  |  |  |
|  |  | **Number** | **Mean (SD)** |
| 1. **Posterior** | **No** | **174,181** | 17·7 (8·2) |
| 1. **Posterior** | **Yes** | **7,336** | 20·0 (8·5) |
| 1. **Lat/Ant-Lat/Hard** | **No** | **88,708** | 17·2 (8·1) |
| 1. **Lat/Ant-Lat/Hard** | **Yes** | **2,592** | 18·4 (8·2) |
| 1. **Ant/Other** | **No** | **11,474** | 18·1 (8·4) |
| 1. **Ant/Other** | **Yes** | **1,211** | 18·1 (8·3) |
| 1. **Trans-trochanteric** | **No** | **658** | 16·0 (7·8) |
|  |  |  | **Comparison** |
|  |  |  | ***Overall:*** *P<0·0001 (regression analysis)* |
|  |  |  | **Pairwise:** |
|  |  |  | ***1 vs 2, 3, 4, 5*** *P<0·001 for each* |
|  |  |  | ***1 vs 6*** *P=0·081* |
|  |  |  | ***1 vs 7*** *P<0·001* |
|  |  |  | ***3 vs 4*** *P<0·001* |
|  |  |  | ***5 vs 6*** *P=0·913* |

**Table S5 Comparison of PROMS OHS scores between the approach subgroups**

| **Approach** | **Minimally invasive procedure used** | **All available**  **pre-operative (Q1) OHS** | | **Complete pairs of pre- (Q1) and post- (Q2) operative OHS** | | | | |
| --- | --- | --- | --- | --- | --- | --- | --- | --- |
|  |  |  |  | **Pre- & post-** | **Pre-**  **(Q1)** | **Post-**  **(Q2)** | **Improvement**  **(=Q2 minus Q1)** | **‘Potential’ change**  **(=48 minus Q1)** |
|  |  | **Number** | **Mean (SD)*** | **Number** | **Mean (SD)*** | **Mean (SD) *Median (IQR)*** | **Mean (SD) *Median (IQR)*** | **Mean (SD)** |
| 1. **Posterior** | **No** | **174,181** | 17·7 (8·2) | **143,439** | 18·2 (8·1) | 39·9 (8·5) *43 (36-46)* | 21·7 (9·8) *22 (15-29)* | 29·8 (8·1) |
| 1. **Posterior** | **Yes** | **7,336** | 20·0 (8·5) | **6,087** | 20·5 (8·4) | 41·4 (7·8) *44 (38-47)* | 20·9 (9·5) *21 (15-27)* | 27·5 (8·4) |
| 1. **Lat/Ant-Lat/Hard** | **No** | **88,708** | 17·2 (8·1) | **72,711** | 17·7 (8·0) | 38·0 (9·3) *41 (33-45)* | 20·3 (10·2) *21 (14-28)* | 30·3 (8·0) |
| 1. **Lat/Ant-Lat/Hard** | **Yes** | **2,592** | 18·4 (8·2) | **2,154** | 18·9 (8·1) | 38·7 (8·8) *41 (34-46)* | 19·8 (10·1) *20 (13-27)* | 29·1 (8·1) |
| 1. **Ant/Other** | **No** | **11,474** | 18·1 (8·4) | **9,468** | 18·6 (8·3) | 39·1 (8·9) *42 (35-46)* | 20·5 (10·1) *21 (14-28)* | 29·4 (8·3) |
| 1. **Ant/Other** | **Yes** | **1,211** | 18·1 (8·3) | **984** | 18·8 (8·2) | 40·7 (8·5) *44 (38-47)* | 21·9 (10·1) *23 (16-29)* | 29·2 (8·2) |
| 1. **Trans-trochanteric** | **No** | **658** | 16·0 (7·8) | **553** | 16·5 (7·9) | 37·4 (9·4) *40 (32-45)* | 20·9 (10·1) *21 (14-28)* | 31·5 (7·9) |
|  |  |  |  |  | **(1)Non-parametric comparison** | |  |  |
|  |  |  |  |  | **Overall:** | *P=0·0001 (Kruskal- Wallis)* |  |  |
|  |  |  |  |  | **Pairwise:** |  |  |  |
|  |  |  |  |  | ***1 vs 2,3,4,5, 6,7*** | *For each P<0·0001 (Dunn’s)* |  |  |
|  |  |  |  |  | ***3 vs 4*** | *P=0·0002* |  |  |
|  |  |  |  |  | ***5 vs 6*** | *P<0·0001* |  |  |
|  |  |  |  |  | **(ii) Parametric comparison (Regression model with adjustment for differences in transformed Q1 values, see text)** | | **(iii) Parametric comparison (Regression model)** |  |
|  |  |  |  |  | **Overall:** | *P<0·0001 (P<0·0001)*** | *P<0·0001 (P<0·0001)*** |  |
|  |  |  |  |  | **Pairwise:** |  |  |  |
|  |  |  |  |  | ***1 vs 2,3,4,5*** | *For each P<0·001 (P<0·001)* | *For each P<0·001 (P<0·001)* |  |
|  |  |  |  |  | ***1 vs 6*** | *P=0·016 (P=0·014)* | *P=0·435 (P=0·442)* |  |
|  |  |  |  |  | ***1 vs 7*** | *P<0·001 (P<0·001)* | *P=0·070 (P=0·074)* |  |
|  |  |  |  |  | ***3 vs 4*** | *P=0·075 (P=0·079)* | *P=0·017 (P=0·019)* |  |
|  |  |  |  |  | ***5 vs 6*** | *P<0·001 (P<0·001)* | *P<0·001 (P<0·001)* |  |

*We reported in an earlier Annual Report (AR2013) that those who *had* a post-op OHS had better pre-op scores. This is reflected here in that the Q1 means are higher when analysis is restricted to those who had **both** Q1 and Q2.

**Comparisons in parenthesis use a ‘robust variance’ approach.

**Table S6 Comparison of the EQ-5D Health Scale (Visual Analogue Score) between the approach subgroups**

| **Approach** | **Minimally invasive procedure used** | **All available**  **pre-operative (Q1) EQ-5D Health Scale** | | **Complete pairs of pre- (Q1) and post- (Q2) operative EQ-5D Health Scale** | | | |
| --- | --- | --- | --- | --- | --- | --- | --- |
|  |  |  |  | **Pre- & post-** | **Pre-**  **(Q1)** | **Post-**  **(Q2)** | **Improvement**  **(=Q2 minus Q1)** |
|  |  | **Number** | **Mean (SD)** | **Number** | **Mean (SD)** | **Mean (SD) *Median (IQR)*** | **Mean (SD)** |
| **(1)Posterior** | **No** | **158,285** | 64·5 (22·0)* | **126,089** | 65·6 (21·5) | 77·4 (17·7)  *80 (70-90)* | - 1. (22·9) |
| **(2)Posterior** | **Yes** | **6,732** | 67·3 (21·3) | **5,416** | 68·4 (20·7) | 79·5 (16·7)  *83 (70-90)* | - 1. (21·9) |
| **(3)Lat/Ant-Lat/Hard** | **No** | **81,022** | 63·8 (22·1) | **64,119** | 64·8 (21·7) | 75·3 (18·2)  *80 (68-90)* | - 1. (23·2) |
| **(4)Lat/Ant-Lat/Hard** | **Yes** | **2,384** | 65·0 (21·3) | **1,904** | 66·2 (20·7) | 76·3 (17·7)  *80 (70-90)* | - 1. (22·6) |
| **(5)Ant/Other** | **No** | **10,465** | 65·3 (21·3) | **8,354** | 66·5 (20·8) | 77·0 (17·6)  *80 (70-90)* | - 1. (22·1) |
| **(6)Ant/Other** | **Yes** | **1,104** | 64·9 (21·7) | **873** | 66·1 (21·3 ) | 78·2 (18·7)  *80 (70-90)* | 1. (23·3) |
| **(7)Trans-trochanteric** | **No** | **595** | 62·4 (22·4) | **487** | 63·7 (22·4) | 75·3 (18·5)  *80 (65-90)* | 11·6 (23·6) |
|  |  |  |  |  | **(i)Non-parametric comparison - unadjusted** | |  |
|  |  |  |  |  | **Overall:** | *P=0·0001 (Kruskal- Wallis)* |  |
|  |  |  |  |  | **Pairwise:** |  |  |
|  |  |  |  |  | ***1 vs 2, 3*** | *For each P<0·0001(Dunn’s)* |  |
|  |  |  |  |  | ***1 vs 4*** | *P=0·0001* |  |
|  |  |  |  |  | ***1 vs 5*** | *P=0·0005* |  |
|  |  |  |  |  | ***1 vs 6*** | *P=0·0140* |  |
|  |  |  |  |  | ***1 vs 7*** | *P=0·0024* |  |
|  |  |  |  |  | ***3 vs 4*** | *P=0·0123* |  |
|  |  |  |  |  | ***5 vs 6*** | *P=0·0008* |  |
|  |  |  |  |  | **(ii) Parametric comparison (Regression model with adjustment for differences in transformed Q1 values**)** | | **(iii) Parametric comparison**  **(Regression model)** |
|  |  |  |  |  | **Overall:** | *P<0·0001 (P<0·0001***)* | *P<0·0001 (P<0·0001***)* |
|  |  |  |  |  | **Pairwise:** |  |  |
|  |  |  |  |  | ***1 vs 2*** | *P<0·001 (P<0·001)* | *P=0·022 (P=0·016)* |
|  |  |  |  |  | ***1 vs 3*** | *P<0·001 (P<0·001)* | *P<0·001 (P<0·001)* |
|  |  |  |  |  | ***1 vs 4*** | *P=0·001 (P=0·001)* | *P=0·001 (P=0·001)* |
|  |  |  |  |  | ***1 vs 5*** | *P<0·001 (P<0·001)* | *P<0·001 (P<0·001)* |
|  |  |  |  |  | ***1 vs 6*** | *P=0·316 (P=0·342)* | *P=0·793 (P=0·796)* |
|  |  |  |  |  | ***1 vs 7*** | *P=0·028 (P=0·033)* | *P=0·820 (P=0·824)* |
|  |  |  |  |  | ***3 vs 4*** | *P=0·068 (P=0·066)* | *P=0·518 (P=0·511)* |
|  |  |  |  |  | ***5 vs 6*** | *P=0·034 (P=0·043)* | *P=0·052 (P=0·055)* |

*Significant differences between group means at Q1 (one-way ANOVA, P<0·001)

**In this model, the best transformation to linearize the relationship between Q1 and Q2 was (1+x)^2^, where x was the Health Scale at Q1

***Comparisons in parenthesis use a ‘robust variance’ approach.

**Table S7 Comparison between the approach subgroups in respect of the responses to the 5 component questions of the PROMs EQ-5D Health Index**

1. **Mobility:**

| **Approach** | **Minimally invasive procedure used** | **Complete pairs of pre- and post-operative question on Mobility** | | | | | | |
| --- | --- | --- | --- | --- | --- | --- | --- | --- |
|  |  | **Pre- & post-**  **Number** | **Pre-op (Q1)** | | | **Post-op (Q2)** | | |
|  |  |  | **No problems**  **in walking about** | **Some problems in walking about** | **Confined to bed** | **No problems**  **in walking about** | **Some problems in walking about** | **Confined to bed** |
| 1. **Posterior** | **No** | **138,839** | 7·0% | 92·8% | 0·3% | 63·4% | 36·6% | 0·1% |
| 1. **Posterior** | **Yes** | **5,907** | 10·6% | 89·2% | 0·2% | 71·2% | 28·7% | 0·1% |
| 1. **Lat/Ant-Lat/Hard** | **No** | **70,633** | 5·7% | 94·0% | 0·3% | 51·4% | 48·5% | 0·1% |
| 1. **Lat/Ant-Lat/Hard** | **Yes** | **2,091** | 8·2% | 91·5% | 0·2% | 55·7% | 44·2% | 0·1% |
| 1. **Ant/Other** | **No** | **9,169** | 7·6% | 92·0% | 0·4% | 58·2% | 41·7% | 0·1% |
| 1. **Ant/Other** | **Yes** | **956** | 8·5% | 91·1% | 0·4% | 65·5% | 34·4% | 0·1% |
| 1. **Trans-trochanteric** | **No** | **545** | 4·0% | 95·6% | 0·4% | 50·3% | 49·7% | 0·0% |
|  |  |  |  |  |  | **Unadjusted comparison of outcome at Q2*** | | |
|  |  |  |  |  |  | **Overall:** | P<0·0001 |  |
|  |  |  |  |  |  | **Pairwise:** |  |  |
|  |  |  |  |  |  | ***1 vs 2, 3, 4, 5*** | *For each P<0·001* |  |
|  |  |  |  |  |  | ***1 vs 6*** | *P=0·177* |  |
|  |  |  |  |  |  | ***1 vs 7*** | *P<0·001* |  |
|  |  |  |  |  |  | ***3 vs 4*** | *P<0·001* |  |
|  |  |  |  |  |  | ***5 vs 6*** | *P<0·001* |  |
|  |  |  |  |  |  | **Comparison of outcome at Q2*, adjusting for Q1** | | |
|  |  |  |  |  |  | **Overall:** | *P<0·0001* |  |
|  |  |  |  |  |  | **Pairwise:** |  |  |
|  |  |  |  |  |  | ***1 vs 2, 3, 4, 5*** | *For each P<0·001* |  |
|  |  |  |  |  |  | ***1 vs 6*** | *P=0·245* |  |
|  |  |  |  |  |  | ***1 vs 7*** | *P<0·001* |  |
|  |  |  |  |  |  | ***3 vs 4*** | *P=0·001* |  |
|  |  |  |  |  |  | ***5 vs 6*** | *P<0·001* |  |

**By logistic regression, after first combining the two outcome categories ‘Some problems’ and ‘confined to bed’ at Q2.*

**(b) Self care**

| **Approach** | **Minimally invasive procedure used** | **Complete pairs of pre- and post-operative question on Self care** | | | | | | |
| --- | --- | --- | --- | --- | --- | --- | --- | --- |
|  |  | **Pre- & post-**  **Number** | **Pre-op (Q1)** | | | **Post-op (Q2)** | | |
|  |  |  | **No problems**  **with self care** | **Some problems washing and dressing** | **Unable to wash or dress myself** | **No problems**  **with self care** | **Some problems washing and dressing** | **Unable to wash or dress myself** |
| 1. **Posterior** | **No** | **138,965** | 46·1% | 52·9% | 1·0% | 81·5% | 18·1% | 0·5% |
| 1. **Posterior** | **Yes** | **5,926** | 54·8% | 44·6% | 0·6% | 86·3% | 13·3% | 0·4% |
| 1. **Lat/Ant-Lat/Hard** | **No** | **70,695** | 44·6% | 54·5% | 1·0% | 79·0% | 20·5% | 0·5% |
| 1. **Lat/Ant-Lat/Hard** | **Yes** | **2,096** | 48·6% | 50·7% | 0·8% | 81·4% | 18·4% | 0·2% |
| 1. **Ant/Other** | **No** | **9,199** | 48·4% | 50·8% | 0·8% | 82·0% | 17·5% | 0·5% |
| 1. **Ant/Other** | **Yes** | **953** | 49·2% | 49·8% | 0·9% | 85·9% | 13·8% | 0·3% |
| 1. **Trans-trochanteric** | **No** | **542** | 38·2% | 60·7% | 1·1% | 74·2% | 25·3% | 0·6% |
|  |  |  |  |  |  | **Unadjusted comparison of outcome at Q2*** | | |
|  |  |  |  |  |  | **Overall:** | *P<0·0001* |  |
|  |  |  |  |  |  | **Pairwise:** |  |  |
|  |  |  |  |  |  | ***1 vs 2, 3*** | *For each P<0·001* |  |
|  |  |  |  |  |  | ***1 vs 4*** | *P=0·888* |  |
|  |  |  |  |  |  | ***1 vs 5*** | *P=0·194* |  |
|  |  |  |  |  |  | ***1 vs 6,7*** | *For each P<0·001* |  |
|  |  |  |  |  |  | ***3 vs 4*** | *P=0·009* |  |
|  |  |  |  |  |  | ***5 vs 6*** | *P=0·003* |  |
|  |  |  |  |  |  | **Comparison of outcome at Q2*, adjusting for Q1** | | |
|  |  |  |  |  |  | **Overall:** | *P<0·0001* |  |
|  |  |  |  |  |  | **Pairwise:** |  |  |
|  |  |  |  |  |  | ***1 vs 2, 3*** | *For each P<0·001* |  |
|  |  |  |  |  |  | ***1 vs 4*** | *P=0·386* |  |
|  |  |  |  |  |  | ***1 vs 5*** | *P=0·933* |  |
|  |  |  |  |  |  | ***1 vs 6,7*** | *For each P=0·001* |  |
|  |  |  |  |  |  | ***3 vs 4*** | *P=0·109* |  |
|  |  |  |  |  |  | ***5 vs 6*** | *P=0·002* |  |

**By logistic regression, after first combining the two outcome categories ‘Some problems washing and dressing’ and ‘unable to wash or dress myself’ at Q2.*

**(c) Usual activity:**

| **Approach** | **Minimally invasive procedure used** | **Complete pairs of pre- and post-operative question on Usual activity** | | | | | | |
| --- | --- | --- | --- | --- | --- | --- | --- | --- |
|  |  | **Pre- & post-**  **Number** | **Pre-op (Q1)** | | | **Post-op (Q2)** | | |
|  |  |  | **No problems**  **with usual activities** | **Some problems with usual activities** | **Unable to perform usual activities** | **No problems**  **with usual activities** | **Some problems with usual activities** | **Unable to perform usual activities** |
| 1. **Posterior** | **No** | **138,495** | 6·2% | 76·1% | 17·7% | 58·9% | 38·3% | 2·8% |
| 1. **Posterior** | **Yes** | **5,889** | 8·6% | 78·2% | 13·2% | 67·0% | 30·8% | 2·2% |
| 1. **Lat/Ant-Lat/Hard** | **No** | **70,383** | 5·4% | 75·8% | 18·8% | 49·6% | 46·5% | 3·9% |
| 1. **Lat/Ant-Lat/Hard** | **Yes** | **2,095** | 6·5% | 76·8% | 16·7% | 52·7% | 43·6% | 3·6% |
| 1. **Ant/Other** | **No** | **9,158** | 7·4% | 75·7% | 16·9% | 55·6% | 41·2% | 3·3% |
| 1. **Ant/Other** | **Yes** | **953** | 6·6% | 78·1% | 15·3% | 62·9% | 34·1% | 3·0% |
| 1. **Trans-trochanteric** | **No** | **542** | 4·2% | 75·8% | 19·9% | 45·9% | 49·6% | 4·4% |
|  |  |  |  |  |  | **Unadjusted comparison of outcome at Q2*** | | |
|  |  |  |  |  |  | **Overall** | *P<0·0001* |  |
|  |  |  |  |  |  | **Pairwise:** |  |  |
|  |  |  |  |  |  | ***1 vs 2, 3, 4, 5*** | *For each P<0·001* |  |
|  |  |  |  |  |  | ***1 vs 6*** | *P=0·013* |  |
|  |  |  |  |  |  | ***1 vs 7*** | *P<0·001* |  |
|  |  |  |  |  |  | ***3 vs 4*** | *P=0·004* |  |
|  |  |  |  |  |  | ***5 vs 6*** | *P<0·001* |  |
|  |  |  |  |  |  | **Comparison of outcome at Q2*, adjusting for Q1** | | |
|  |  |  |  |  |  | **Overall** | *P<0·0001* |  |
|  |  |  |  |  |  | **Pairwise:** |  |  |
|  |  |  |  |  |  | ***1 vs 2, 3, 4, 5*** | *For each P<0·001* |  |
|  |  |  |  |  |  | ***1 vs 6*** | *P=0·031* |  |
|  |  |  |  |  |  | ***1 vs 7*** | *P<0·001* |  |
|  |  |  |  |  |  | ***3 vs 4*** | *P=0·021* |  |
|  |  |  |  |  |  | ***5 vs 6*** | *P<0·001* |  |

**By logistic regression, after first combining the two outcome categories ‘Some problems with usual activities’ and ‘unable to perform usual activities’ at Q2.*

**(d) Pain/discomfort:**

| **Approach** | **Minimally invasive procedure used** | **Complete pairs of pre- and post-operative question on Pain/discomfort** | | | | | | |
| --- | --- | --- | --- | --- | --- | --- | --- | --- |
|  |  | **Pre- & post-**  **Number** | **Pre-op (Q1)** | | | **Post-op (Q2)** | | |
|  |  |  | **No pain or discomfort** | **Moderate pain or discomfort** | **Extreme pain or discomfort** | **No pain or discomfort** | **Moderate pain or discomfort** | **Extreme pain or discomfort** |
| 1. **Posterior** | **No** | **136,360** | 0·7% | 58·0% | 41·2% | 58·6% | 38·3% | 3·2% |
| 1. **Posterior** | **Yes** | **5,791** | 1·1% | 66·5% | 32·4% | 64·9% | 32·5% | 2·6% |
| 1. **Lat/Ant-Lat/Hard** | **No** | **69,406** | 0·6% | 55·3% | 44·1% | 50·7% | 45·4% | 4·0% |
| 1. **Lat/Ant-Lat/Hard** | **Yes** | **2,055** | 0·9% | 60·5% | 38·6% | 53·4% | 42·9% | 3·8% |
| 1. **Ant/Other** | **No** | **9,020** | 0·7% | 59·3% | 40·0% | 54·8% | 41·4% | 3·8% |
| 1. **Ant/Other** | **Yes** | **950** | 0·8% | 61·1% | 38·1% | 60·8% | 35·5% | 3·7% |
| 1. **Trans-trochanteric** | **No** | **531** | 0·0% | 50·7% | 49·3% | 46·5% | 49·3% | 4·1% |
|  |  |  |  |  |  | **Unadjusted comparison of outcome at Q2*** | | |
|  |  |  |  |  |  | **Overall** | *P<0·0001* |  |
|  |  |  |  |  |  | **Pairwise:** |  |  |
|  |  |  |  |  |  | ***1 vs 2, 3, 4, 5*** | *For each P<0·001* |  |
|  |  |  |  |  |  | ***1 vs 6*** | *P=0·158* |  |
|  |  |  |  |  |  | ***1 vs 7*** | *P<0·001* |  |
|  |  |  |  |  |  | ***3 vs 4*** | *P=0·016* |  |
|  |  |  |  |  |  | ***5 vs 6*** | *P<0·001* |  |
|  |  |  |  |  |  | **Comparison of outcome at Q2*, adjusting for Q1** | | |
|  |  |  |  |  |  | **Overall:** | *P<0·0001* |  |
|  |  |  |  |  |  | **Pairwise:** |  |  |
|  |  |  |  |  |  | ***1 vs 2, 3, 4, 5*** | *For each P<0·001* |  |
|  |  |  |  |  |  | ***1 vs 6*** | *P=0·257* |  |
|  |  |  |  |  |  | ***1 vs 7*** | *P<0·001* |  |
|  |  |  |  |  |  | ***3 vs 4*** | *P=0·098* |  |
|  |  |  |  |  |  | ***5 vs 6*** | *P<0·001* |  |

**By logistic regression, after first combining the two outcome categories ‘Moderate pain or discomfort’ and ‘Extreme pain or discomfort’ at Q2.*

**(e) Anxiety/depression:**

| **Approach** | **Minimally invasive procedure used** | **Complete pairs of pre- and post-operative question on Anxiety/depression** | | | | | | |
| --- | --- | --- | --- | --- | --- | --- | --- | --- |
|  |  | **Pre- & post-**  **Number** | **Pre-op (Q1)** | | | **Post-op (Q2)** | | |
|  |  |  | **Not**  **anxious or depressed** | **Moderately anxious or depressed** | **Extremely anxious or depressed** | **Not**  **anxious or depressed** | **Moderately anxious or depressed** | **Extremely anxious or depressed** |
| 1. **Posterior** | **No** | **137,759** | 59·7% | 36·0% | 4·4% | 83·4% | 15·1% | 1·6% |
| 1. **Posterior** | **Yes** | **5,865** | 61·8% | 34·9% | 3·3% | 86·2% | 12·6% | 1·2% |
| 1. **Lat/Ant-Lat/Hard** | **No** | **69,895** | 58·1% | 37·2% | 4·7% | 80·2% | 17·9% | 1·9% |
| 1. **Lat/Ant-Lat/Hard** | **Yes** | **2,078** | 59·3% | 36·0% | 4·7% | 82·6% | 16·0% | 1·4% |
| 1. **Ant/Other** | **No** | **9,096** | 59·2% | 36·5% | 4·4% | 82·0% | 16·2% | 1·9% |
| 1. **Ant/Other** | **Yes** | **949** | 62·4% | 34·0% | 3·6% | 84·8% | 13·9% | 1·3% |
| 1. **Trans-trochanteric** | **No** | **538** | 62·6% | 33·1% | 4·3% | 82·9% | 14·5% | 2·6% |
|  |  |  |  |  |  | **Unadjusted comparison of outcome at Q2*** | | |
|  |  |  |  |  |  | **Overall:** | P<0·0001 |  |
|  |  |  |  |  |  | **Pairwise:** |  |  |
|  |  |  |  |  |  | ***1 vs 2, 3*** | For each P<0·001 |  |
|  |  |  |  |  |  | ***1 vs 4*** | P=0·378 |  |
|  |  |  |  |  |  | ***1 vs 5*** | P=0·001 |  |
|  |  |  |  |  |  | ***1 vs 6*** | P=0·225 |  |
|  |  |  |  |  |  | ***1 vs 7*** | P=0·778 |  |
|  |  |  |  |  |  | ***3 vs 4*** | P=0·007 |  |
|  |  |  |  |  |  | ***5 vs 6*** | P=0·029 |  |
|  |  |  |  |  |  | **Comparison of outcome at Q2*, adjusting for Q1** | | |
|  |  |  |  |  |  | **Overall:** | P<0·0001 |  |
|  |  |  |  |  |  | **Pairwise:** |  |  |
|  |  |  |  |  |  | ***1 vs 2, 3*** | For each P<0·001 |  |
|  |  |  |  |  |  | ***1 vs 4*** | P=0·464 |  |
|  |  |  |  |  |  | ***1 vs 5*** | P=0·001 |  |
|  |  |  |  |  |  | ***1 vs 6*** | P=0·546 |  |
|  |  |  |  |  |  | ***1 vs 7*** | P=0·437 |  |
|  |  |  |  |  |  | ***3 vs 4*** | P=0·011 |  |
|  |  |  |  |  |  | ***5 vs 6*** | P=0·115 |  |

**By logistic regression, after first combining the two outcome categories ‘Moderately anxious or depressed’ and ‘Extremely anxious or depressed’ at Q2.*

**Table S8 Other PROMS - comparison of patient self-report post-operative (Q2) problems between the approach subgroups·**

| **Approach** | **Minimally invasive procedure used** | **Did you experience any of the following problems after your operation? (Y/N)** | | | | **Have you been readmitted to hospital since the operation on your hip?** | **Have you had another operation on your hip since your replacement operation?** |
| --- | --- | --- | --- | --- | --- | --- | --- |
|  |  | **Allergy or reaction to drug?** | **Urinary problems?** | **Bleeding?** | **Wound problems?** |  |  |
| **(1)Posterior** | **No** | 14,951/136,887  **(10·9%)** | 16,891 /135,162  **(12·5%)** | 5,756/132,678  **(4·3%)** | 10,443/134,460  **(7·8%)** | 9,557/144,623  **(6·6%)** | 2,791/144,706  **(1·9%)** |
| **(2)Posterior** | **Yes** | 642/5,827  **(11·0%)** | 768/5,750  **(13·4%)** | 221/5,649  **(3·9%)** | 290/5,689  **(5·1%)** | 330/6,100  **(5·4%)** | 108/6,112  **(1·8%)** |
| **(3)Lat/Ant-Lat/Hard** | **No** | 8,114/68,292 **(11·9%)** | 9,150/67,365  **(13·6%)** | 3,664/66,059  **(5·6%)** | 7,452/67,621  **(11·0%)** | 5,245/73,309  **(7·2%)** | 1,443/73,374  **(2·0%)** |
| **(4)Lat/Ant-Lat/Hard** | **Yes** | 214/2,023  **(10·6%)** | 273/2,013  **(13·6%)** | 97/1,969  **(4·9%)** | 186/2,009  **(9·3%)** | 141/2,159  **(6·5%)** | 36/2,163  **(1·7%)** |
| **(5)Ant/Other** | **No** | 959/8,942  **(10·7%)** | 1,126/8,833  **(12·8%)** | 374/8,640  **(4·3%)** | 857/8,818  **(9·7%)** | 706/9,525  **(7·4%)** | 206/9,527  **(2·2%)** |
| **(6)Ant/Other** | **Yes** | 99/961  **(10·3%)** | 106/949  **(11·2%)** | 40/942  **(4·3%)** | 67/947  **(7·1%)** | 54/1,002  **(5·4%)** | 11/1,002  **(1·1%)** |
| **(7)Trans-trochanteric** | **No** | 54/519  **(10·4%)** | 55/512  **(10·7%)** | 30/513  **(5·9%)** | 64/526  **(12·2%)** | 25/563  **(4·4%)** | 5/562  **(0·9%)** |
| **Group comparison (logistic regression)** | |  |  |  |  |  |  |
| **Overall:** | | *P<0·0001* | *P<0·0001* | *P<0·0001* | *P<0·0001* | *P<0·0001* | *P=0·0409* |
| **Pairwise:** | |  |  |  |  |  |  |
| ***1 vs 2**** | | *P=0·819* | *P=0·054* | *P=0·123* | *P<0·001* | *P<0·001* | *P=0·367* |
| ***1 vs 3*** | | *P<0·001* | *P<0·001* | *P<0·001* | *P<0·001* | *P<0·001* | *P=0·544* |
| ***1 vs 4*** | | *P=0·623* | *P=0·152* | *P=0·204* | *P=0·013* | *P=0·886* | *P=0·375* |
| ***1 vs 5*** | | *P=0·562* | *P=0·490* | *P=0·966* | *P<0·001* | *P=0·002* | *P=0·110* |
| ***1 vs 6*** | | *P=0·539* | *P=0·218* | *P=0·890* | *P=0·428* | *P=0·122* | *P=0·060* |
| ***1 vs 7*** | | *P=0·706* | *P=0·231* | *P=0·095* | *P<0·001* | *P=0·040* | *P=0·081* |
| ***3 vs 4*** | | *P=0·074* | *P=0·979* | *P=0·236* | *P=0·013* | *P=0·267* | *P=0·318* |
| ***5 vs 6*** | | *P=0·687* | *P=0·164* | *P=0·906* | *P=0·009* | *P=0·019* | *P=0·027* |

****Pairwise odds-ratios available if needed***
